# XAS Data Preprocessing of Nanocatalysts for Machine Learning Applications

**DOI:** 10.3390/ma14247884

**Published:** 2021-12-20

**Authors:** Oleg O. Kartashov, Andrey V. Chernov, Dmitry S. Polyanichenko, Maria A. Butakova

**Affiliations:** The Smart Materials Research Institute, Southern Federal University, 178/24 Sladkova, 344090 Rostov-on-Don, Russia; cherno@sfedu.ru (A.V.C.); codeconcil@gmail.com (D.S.P.); mbutakova@sfedu.ru (M.A.B.)

**Keywords:** functional materials, materials characterization, data preprocessing, X-ray absorption spectra, machine learning

## Abstract

Innovative development in the energy and chemical industries is mainly dependent on advances in the accelerated design and development of new functional materials. The success of research in new nanocatalysts mainly relies on modern techniques and approaches for their precise characterization. The existing methods of experimental characterization of nanocatalysts, which make it possible to assess the possibility of using these materials in specific chemical reactions or applications, generate significant amounts of heterogeneous data. The acceleration of new functional materials, including nanocatalysts, directly depends on the speed and quality of extracting hidden dependencies and knowledge from the obtained experimental data. Usually, such experiments involve different characterization techniques and different types of X-ray absorption spectroscopy (XAS) too. Using the machine learning (ML) methods based on XAS data, we can study and predict the atomic-scale structure and another bunch of parameters for the nanocatalyst efficiently. However, before using any ML model, it is necessary to make sure that the XAS raw experimental data is properly pre-processed, cleared, and prepared for ML application. Usually, the XAS preprocessing stage is vaguely presented in scientific studies, and the main efforts of researchers are devoted to the ML description and implementation stage. However, the quality of the input data influences the quality of ML analysis and the prediction results used in the future. This paper fills the gap between the stage of obtaining XAS data from synchrotron facilities and the stage of using and customizing various ML analysis and prediction models. We aimed this study to develop automated tools for the preprocessing and presentation of data from physical experiments and the creation of deposited datasets on the basis of the example of studying palladium-based nanocatalysts using synchrotron radiation facilities. During the study, methods of preliminary processing of XAS data were considered, which can be conditionally divided into X-ray absorption near edge structure (XANES) and extended X-ray absorption fine structure (EXAFS). This paper proposes a software toolkit that implements data preprocessing scenarios in the form of a single pipeline. The main preprocessing methods used in this study proposed are principal component analysis (PCA); z-score normalization; the interquartile method for eliminating outliers in the data; as well as the k-means machine learning method, which makes it possible to clarify the phase of the studied material sample by clustering feature vectors of experiments. Among the results of this study, one should also highlight the obtained deposited datasets of physical experiments on palladium-based nanocatalysts using synchrotron radiation. This will allow for further high-quality data mining to extract new knowledge about materials using artificial intelligence methods and machine learning models, and will ensure the smooth dissemination of these datasets to researchers and their reuse.

## 1. Introduction

### 1.1. Problem Formulation

**Figure 1:**
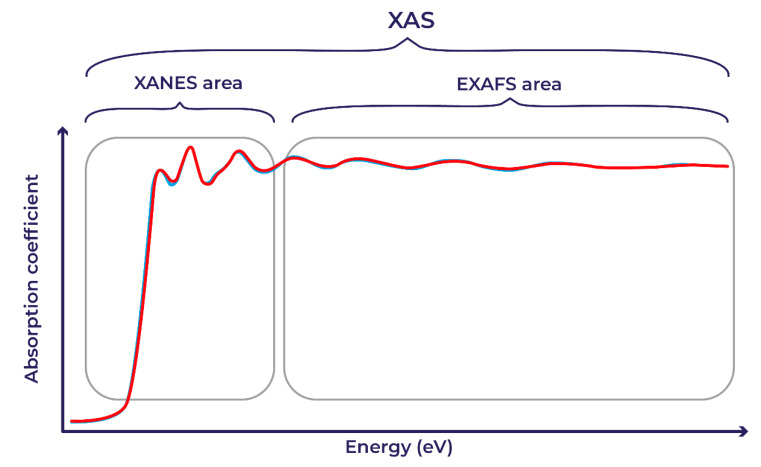
XANES and EXAFS areas within XAS.

Nanocatalysis in the design of new functional materials is an important and integral part of both academic research and in applications to modern industry. The obtained scientific results in the field of nanocatalysis allow a new look at the organization of technical processes in the energy and chemical industries. The innovative development of these companies is currently relevant and promising in the world [1]. One of the main precise methods for studying nanocatalysts are in situ/operando methods using synchrotron radiation [2]. In this case, the key objects of interest are the local geometric and electronic structures of the material. Different variants of X-ray absorption spectroscopy (XAS) in various synchrotron radiation facilities are frequently used to characterize the properties [3,4] at the nanoscale. XAS examines the interaction of a substance with photons with energies in the X-ray range (from hundreds of eV to hundreds of keV), while the range used in each particular experiment is selected in accordance with the binding energy of core electrons in atoms and the local environment of interest. When performing X-ray absorption experiments, the experimenters are primarily interested in measuring the measurement of absorption coefficient, μ, which shows the relationship between the intensity of X-rays entering a material sample; the thickness of the material sample; and the intensity of the beam passed through the material sample. This relationship is usually described with the Beer–Lambert–Bouguer law. For most X-ray energies, the absorption coefficient μ is a smooth function of energy with a value that depends on the sample density ρ, atomic number Z, the atomic mass M, and X-ray energy E: μ ≈ (ρ Z^4^)/(ME^3^). However, there are materials in which the monotonic change in the absorption coefficient is disturbed, for example, Pd-based nanocatalysts with active 3D centers made from much lighter chemical elements. Because of this fact, the entire XAS area (an example is shown in Figure 1) can be conditionally divided into two areas [5,6]: X-ray absorption near edge structure (XANES) and extended X-ray absorption fine structure (EXAFS).

For nanocatalysts, XANES is used to quantify the oxidation state, and EXAFS makes it possible to determine interatomic distances, coordination numbers, and amplitudes of thermal deviations. XAS data allow [7] the characterization of new catalyst types and determining the dynamics of changes in the structure of materials during various chemical reactions. This fact further allows the extraction of structure–property relationships from experimental data, which is of decisive importance for the discovery of a wide range of new functional materials [8].

In the last decade, significant progress has been made in the theory and applications of spectroscopy techniques, including various machine learning (ML) approaches. New types and possibilities of physics experiments, an increase in their resolution, an increase in data flows, and the speed of their collection [9], are becoming excellent prerequisites for the creation of methods for accurate, fast, and effective data analysis [10]. However, the solution of this problem requires detailed theoretical calculations, high-quality preliminary processing of the obtained data of physical experiments, their convenient presentation for the purpose of searching for the physical dependencies that underlie them, reuse of the collected data, and their reproducibility. The importance of reusing these experiments is excellently demonstrated in this study [11].

The aim of this study is to propose an approach for the preliminary assessment and processing of XAS data obtained from synchrotron radiation facilities, which would allow further application of ML models for the accelerated design of new nanocatalysts.

### 1.2. Mini Review of Existing Solutions

The meaning of data preprocessing is mostly the extent in transforming the initial data into more suitable form for solving specific problems. Frequently, at this stage, the extraction of resources in the physical subject area is not necessary, it is required to find suitable methods for optimizing the dimension, transforming data without noticeable loss in information content for more efficient processing and extraction of the hidden dependences at the subsequent stages of data mining. Data preprocessing is not required outside the context of using for the further results obtained. Based on this, that is a means to achieve the goal, in our case, transforming datasets to a specific form while preserving information content. Hence, it cannot be argued that in all sets of tasks their possible application of standard methods or methodology for transforming data from the original set to the required one. Additionally, the processes of collecting, aggregating, and preprocessing data often claim a significant part of research resources needed at is required to extract new knowledge subject area of research. All of the above shows the significance of data preprocessing both for their reuse in scientific research and for more efficient and high-quality operation at later stages of scientific production.

One of the most common tools for data analysis using chemometric methods is the MATLAB software package. It is great for preprocessing chemometric data thanks to its integrated capabilities, for example, functions implementation normalizing XAS spectra to the absorption range 0 to 1 or converting spectral absorption to transmittance, and more. Sure, the clear advantages of this product include the wide possibilities of data visualization and integration into the preprocessing pipeline of preliminary processing of machine learning methods, which significantly speed up the process [12], but its use is limited to the platform, which does not always allow for the consideration and modifying computational processes for the required level of detail.

There are many software solutions that partially automate data preprocessing and analysis—such as FEFFIT, IFEFFIT, XDAP, EXAFSPAK, WINXAS, XFIT, EXAFS Data Analysis (EDA), xTunes, MAX. The most popular are the FEFF and IFEFFIT libraries, as well as software complexes based on them, such as ATHENA (not Amazon Athena), where the main functionality is devoted to data processing issues, ARTEMIS is a solution for EXAFS data analysis based on standards of FEFF [13], HEPHAESTUS is a set of utilities for interacting with the beamline based on tabular data [14]. They compare favorably with the form of licenses for distributed software tools, the possibilities of fitting physical spectra data, tools for graphical display of XAFS data, and—in general—a powerful set of functionalities for high-quality automated analysis of data from physical experiments using synchrotron radiation. In addition, FEFFIT’s XAFS data automatic modeling capabilities provide a wide range of additional capabilities. XDAP is an easy-to-use program for the complete analysis of X-ray absorption spectra, the functionality of which is very similar to the solutions discussed above [15].

A distinctive feature of EXAFSPAK (developed by Dr. J.N. George from SSRL) is the ability to use the ab initio principle of multiple scattering from FEFF when plotting curves, which provides a faster result [16]. Another data analysis tool is the free software WINXAS, which contains all the common functions for processing XAFS data together with a set of common numerical procedures [17]. The EXAFS Data Analysis software package, called EDA, allows you to perform all stages of the extended X-ray absorption fine structure (EXAFS) analysis and obtain structural information about the local environment around the absorbing center [18]. A tool for detailed and operational analysis of XAS data, which, in addition to basic processing of experimental spectra data, can visualize them in real time with pre-processed XAS parameters [19]. Additionally, multiplatform applications for XAFS (MAX), consisting of four modules, 2 of which are devoted to the issue under consideration; in particular, CHEROKEE is responsible for processing the EXAFS and XANES data, and CRYSTALFFREV allows theoretical calculations of FEFF EXAFS and XANES [20]. All the considered tools have powerful functionality for analyzing experimental and modeling theoretical XAS data, however, they are enclosed in clearly established boundaries, normalized by the main types of calculations performed and finding the key characteristics of materials. The data preprocessing module is often partially automated; moreover, the result obtained cannot always be examined outside the context of the functionality of software packages. Furthermore, the review work [21] proposes various theoretical methods for analyzing EXAFS data to characterize the catalysts under study, which primarily aimed at the direct extraction of primary data and material characteristics. Basically, most of the works are directed towards the analysis of the data obtained, without going into the details of their preliminary processing, which in the future can lead to difficulties in the reproducibility of the result obtained or its reuse in other studies. Separately, the web platform SPECTRA (DEVAS) should be highlighted, which implements convenient functionality for the study and visualization of data from the spectra of physical experiments [22]. In addition, this product can be distinguished from others due to its wide possibilities for the reuse of experimental data.

Recently, researchers are increasingly using machine learning methods to process and analyze data from XANES and EXAFS spectra. The motivation for the application of artificial intelligence and machine learning algorithms is to reduce the computational complexity of analysis algorithms; to reveal new possibilities for extracting hidden dependence, for example, the relationship of structure-property; etc. Therefore, in the work [23] proposed of the PyFitit software, in which several machine learning algorithms such as gradient boosting, random forest, radial basis function, and neural networks were implemented. They are used to refine the structure of a material using X-ray spectra. As preliminary stages of the proposed intellectual analysis of the author, statistical criteria were used to determine the amount of the main components with the subsequent restoration of pure spectral profiles, using the method of principal components, approximated by a theoretical interpolated spectrum. In [5], the authors reported on the creation of a new spectra matching algorithm—ensemble-learned spectra identification (ELSIE)—using the ensemble learning method to increase the stability of XAS, as well as a database of calculated spectra of reference X-ray radiation.

In general, most of the works devoted to the methods of analysis and processing of XANES and EXAFS spectra data are aimed at extracting exclusively knowledge about new materials and achieving incredible results in the physical field of research, but without disclosing the methods of collecting and preprocessing, for example [24,25].

## 2. Materials and Methods

### 2.1. Retrieving Source Datasets

**Figure 2:**
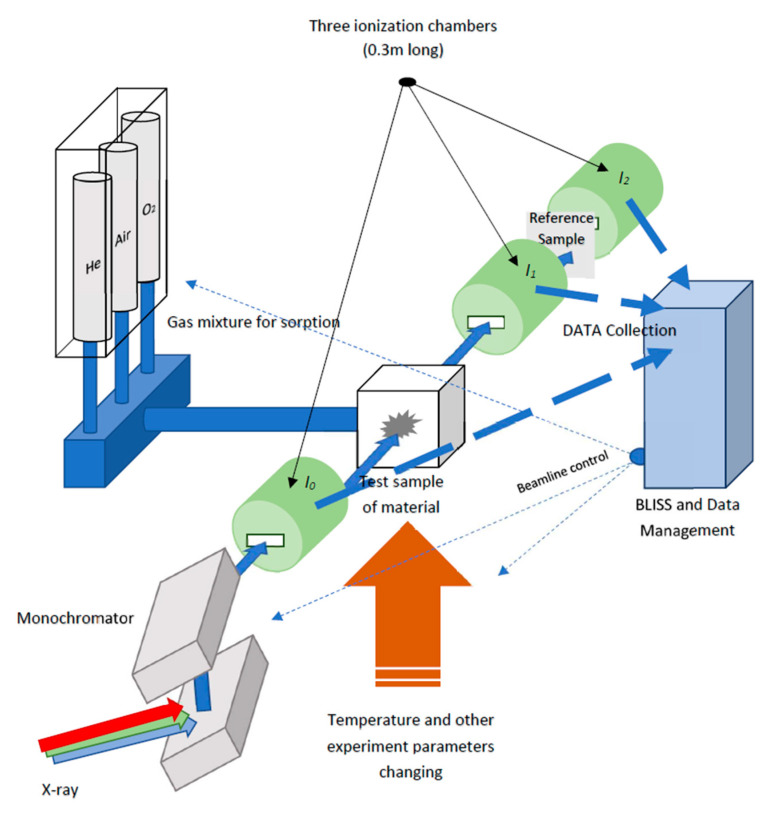
General scheme for data collection from ESRF BM31 beamline.

XAS data for this study were collected during a long period of time from 2013 to 2021 from experiments with Pd-based nanocatalysts and include 8283 XAS spectra. In terms of time, the raw XAS data are not balanced because approximately 7500 XAS spectra were obtained in 2021. Data collection was carried out at the European Synchrotron Radiation Facility (ESRF) [26], Grenoble, France. The experiments used three ESRF beamlines: BM01b, BM26A, and BM31. Since, in fact, BM01b is analogous to the BM31 beamline, we can conditionally divide them into two channels used to receive XAS data, and in the next text, BM01b and BM31 beamlines can be considered as one and the same beamline. The beamline BM26A is mostly available for Collaboration Research Groups. XAS experiments using this line are mainly specialized mainly in real-time catalysis studies [27]. We collected our XAS data from the Swiss–Norwegian beamline BM31 (BM01b) under different experimental conditions studying Pd-based nanocatalysts. This beamline is beneficial for the experiments under consideration on its technical characteristics and functionality. The BM31 beamline provided the ability step-by-step collection of experimental data with a time interval of 1 s, fast scanning of the monochromator for fast XANES measurements, and a wide range of energies (4.9–70 keV) [28,29]. The general scheme of the data collection from BM31 is presented in Figure 2. Here, the ionization chambers are marked in green, at the output of which we obtain the values of the currents I_0_, I_1_, I_2_, generated based on the quantitative characteristics of the X-ray transmission, which are subsequently converted into the absorption coefficient.

The proprietary ESRF software, BeamLine Instrumentation Support Software (BLISS), was used to control the beams and record experimental data from 2016 to 2021. Previously, SPEC was used, providing three specialized functions for communicating with the TACO distributed server system and ESRF-developed drivers developed by ESRF for controlling equipment. BLISS is essentially a software package with a set of Python tools and libraries. It is represented as a Python extension bundle, allowing it to be integrated into existing Python applications. In addition, such a structure is a convenient and flexible way to write tools for the main functions of a project. There are two user interfaces that allow researchers or engineers to access BLISS functions [30]. BLISS has excellent built-in data management tools; all objects are assigned unique names to clearly identify the data sources. The BLISS data model is built from the bottom up, which is convenient for conducting experiments. When we collected XAS data from our experiments, it is placed in the channels supporting message transfer between BLISS sessions, from where it is published in the Redis database, where it is stored during the configuration time of the parameter values. BLISS also includes a Python helper for scanning and presenting the collected data.

### 2.2. Suggested Methods and Algorithms

Today, the information technologies and capability of computational tools of research allow generating a huge amount of data for varying degrees of detail of the ongoing processes in various scientific areas. Therefore, the subsequent discovering new facts and knowledge requires an organized and structured approach to processing them. Most of the data received is also either partially structured information or completely unstructured. To reduce the volumes of the studied data without losing their information content, as well as to bring them into a convenient one for further analysis format, their preliminary processing is necessary. Thus, the correct choice of the preprocessing stages of approach preprocessing and prepared datasets can increase the speed of the mining process for the extraction of new facts and knowledge [31].

**Figure 3:**
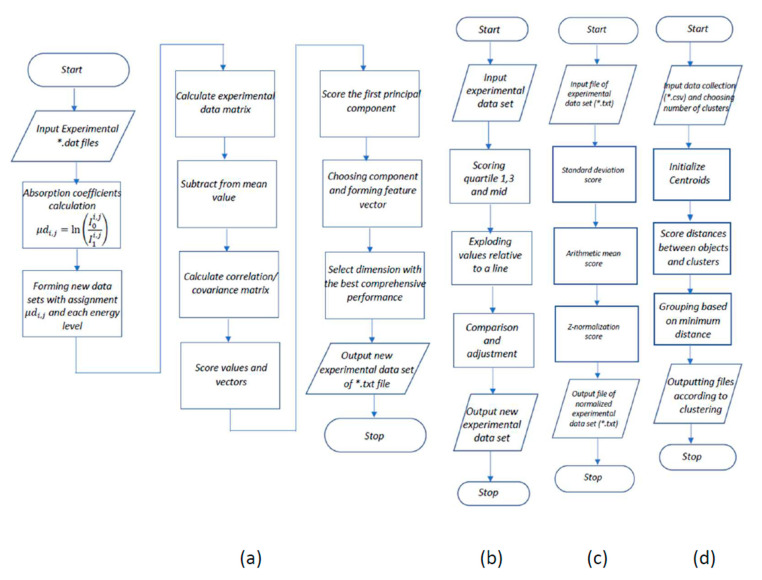
Flowcharts of algorithms for the proposed methods. (**a**)—PCA algorithm; (**b**)—Interquartile method algorithm; (**c**)—Z-score normalization algorithm; (**d**)—K-means clustering algorithm.

The methods that we used during this study were aimed at the development of automated software for the preliminary processing and presentation of the XAS data for the development of nanocatalysts based on Pd nanoparticles further assisted by ML. To solve this problem, several well-known methods were used to interpolate, improve (featurization), normalize, and eliminate statistical outliers in the data. The algorithms of the proposed methods are shown in Figure 3. In the cases when a series of physical experiments with the use of synchrotron radiation is carried out and which results in series of spectra, it can be argued that it is possible to reflect all these processes with part of the spectra without losing data.

In particular, to interpolate the obtained XAS absorption spectra data near the region of the edge structure, the principal component analysis was used to obtain the values of the normal distribution of the absorption coefficient relative to the incoming energy. The basis for the software implementation of the proposed approach was the research results presented in the papers [32,33,34]. Principal component analysis (PCA) implements mathematical operations that interpolate raw data from physical experiments by transforming a series of correlated observations into a smaller set of uncorrelated observations, called principal components, without significant loss of information content of the dataset. The first major component represents the greatest possible data variability. Subsequent components describe the remaining variability [35]. In our case, interpolation means replacing a complex spectral dependence function from a series of experiments with a simpler one, which will give a certain increase in speed. To solve the problem of feature selection, the feature extraction approach was used, since in our case, each experimental observation makes an equal contribution to the assessment of information content. Cross-validation was used to evaluate the resulting model and reinforce the choice of number of components [36]. The flowchart of this method and algorithm is given in part (a) in Figure 3. The data normalization function was implemented using the Z normalization approach [37], the algorithm of the software implementation of which is given in part (c) in Figure 3.

The algorithm of the proposed software toolkit for preprocessing the XAS data from experiments is quite voluminous, therefore the pseudocode of the algorithm is avaliable in the Data Availability Statement (“CSVParserPseudocode”), where the normalization class is designated as “Normalization”. This class is based on the use of three methods, in particular, “AverageValue”—calculating the arithmetic mean of the data range within each experiment, “MeanSquareDeviation”—responsible for calculating the standard deviation of one measurement within the experiment from the arithmetic mean and “ZNormalize”—calculating the Z normalization of the data in question. The “Emissions” class is responsible for finding and eliminating statistical outliers using the software implementation of the statistical interquartile method for finding outliers in the data [38]. The CSVParser class is responsible for merging data from the result files (* txt file format) and experiment parameters (* param file format) by parsing the given data to match and extract the required information using regular expressions. The pseudocode of the algorithm of the proposed class is shown in Algorithm 1.
**Algorithm 1** Parse data and make output files [CSVParser(txtSourseFilePath, paramSourseFilePath)].**INPUT:** txt file with energy data and mod, param file with observation data**OUTPUT:** csv file with combined dataframe of observationstxtFile = Open(txtSourseFilePath)paramFile = Open(paramSourseFilePath)**while** !endOfData(txtFile) **or** !endOfData(paramFile)   txtFields = readFields(txtFile);   paramFields = readFields(paramFile);    **if** first       txtHeader = txtFields;       paramwHeader = paramFields;    **for** i = 0 **to** i < Length(paramFields)      combinedData[paramHeader[i]] = paramFields[i];    **for** i = 0 **to** i < Length(txtFields)      combinedData[txtHeader[i]] = txtFields[i];    resultFile.Write(combinedData);**return** resultFile;

The extracted values are separated from each other by adding a tab delimiter to represent the resulting file in comma-separated values (* CSV) format. The symbol “,” was used as a separator. In addition, the methods “FindRepeatLine” and “FindEmptyField” were implemented, which allow eliminating duplicate lines, as well as searching and filling in empty fields within the results of experimental measurements.

The software implementation of the proposed approach is available at https://github.com/codeConcil/Sfedu_csv_parser (accessed on 11 December 2021). Software implementations of the classes are located in “Sfedu_csv_parser/CSVParser/Refactoring/”, where “CSVRefactor.cs” and “CSVRefactorClassificate.cs” are responsible for complete data merging and data merging according to the type of the used phase state of the investigated material, respectively. “CSVRefactorEmissionsAnomaly.cs” is a class for finding and eliminating outliers in data within a single experiment in a series. “CSVRefactorFindEmpty.cs”, “CSVRefactorFindNone.cs”, and “CSVRefactorFindRepeat.cs” are classes for finding and filling empty fields in data structures, and finding and removing repeated experiments in a series (if any). The “CSVRefactorNormalizationAnomaly.cs” class that implements z-score normalization for the selected range of given data.

In some cases, in scrutinized data sets, the phase of the sample material can be determined inaccurately. This is due to the impossibility of determining the quantitative characteristics of the materials included in the sample during the process flow of a chemical reaction. For a more accurate separation of the phases of a substance relative to the data of the experiments, it is proposed to cluster observations of the available data sets. It is proposed to use the K-means machine learning algorithm as a clustering method. K-means clustering is a precise clustering technique that attempts to cluster data by grouping related attributes into uniquely defined clusters. Each data point in a dataset is assigned to only single cluster. When data are split, only cluster centers are moved and the state of all data points is captured. Clustering is an iterative process to find the best and best cluster centers. Distance metrics determined how far a point is from the center of a cluster [39]. The description of this algorithm is shown in Figure 3—Part (d). The use of cluster analysis allows us to more accurately indicate the phase of the sample under study founded on the rest of the experimental data. In our case, the rows of the table are fed to the input of the algorithm, corresponding to the experiment being carried out under the given conditions, converted into vectors of features, with which the algorithm works. Additionally, this clustering algorithm was chosen based on the principle of its operation—the initial choice of the number of clusters, which in our case allows for a more efficient implementation of the strategy for specifying the parameters of physical experiments. The principle of operation is simple at one of the final stages of preliminary processing, we cluster the data and if, for example, an experiment with the phase state of a substance a metal is clustered into oxides, then a metal/oxide marker is added in the column with the phase state of the substance.

## 3. Results

### 3.1. Structure of the Datasets under Study

**Figure 4:**
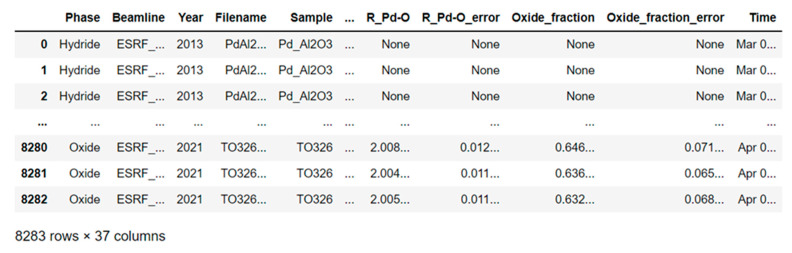
Example of content of XAS experiments PARAM file.

One of the files created as a result of each of our XAS experiments in the series is a file with the PARAM extension. This file type is a text file and does not have tab delimiters in its structure. The PARAM content is formed from the values of the line configuration parameters and the experiment being performed. These values are unique initial test input data and can be set both automatically for static parameters and manually, for example, to specify the angle of rotation of the monochromator. An example of the content of one such file is shown in the Figure 4.

A total of 37 columns in this file contain the initial conditions of the XAS experiment. The first column indicates the phase state of the sample of palladium nanoparticles used, so in our case, we are dealing with four different phases of the substance—hydride, carbide, metal, and oxide. When refining the quantitative indicators of the use of various phases of palladium, we found that the oxide phase was the most common in the research—7108 measurements, and the rarest was carbide—only 14 measurements. The phases of the metal and hydride are presented in intermediate values at the level of about 500 measurements for each. The next three columns indicate the synchrotron radiation output channel used, the year of the experiment, and the name of the file with the direct measurement results. The values of the next two columns indicate the names of the samples that were taken as reference values and obtained values, respectively. In the seventh column of the parameter file, the temperature value is indicated for each individual experiment; in general, the data are used to demonstrate a fairly wide temperature range. The next eight columns display exactly which gases, or their mixtures, were selected for the sorption of the catalyst.

**Figure 5:**
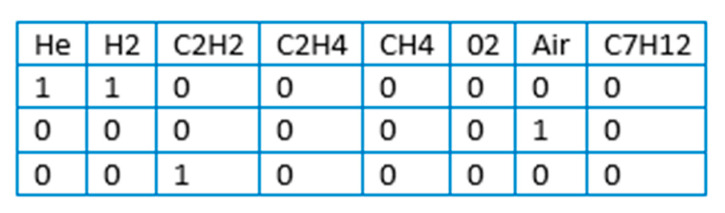
Example of indicators of gases used in XAS experiment.

The total set of gases is expressed by the following elements: He, H_2_, C_2_H_2_, C_2_H_4_, CH_4_, O_2_, air mixture, C_7_H_12_ with a binary indication of the objects involved, where “1” was used to purge Pd nanoparticles in this particular experiment, “0” was not used (Figure 5).

The last column of the file records the exact date and time of the experiment. The remaining parameters given in the halyard are responsible for the local geometric and electronic structure of the material, including coordination numbers, bonds and their angles, interatomic distances, amplitude, inclusions, etc. In particular, the following three parameters with the headings “Amp_ref”, “Amp_ref_error”, and “Amp_spec” indicate the amplitude and its error for the reference sample and for the nanocatalyst, respectively. The columns “N_Pd-Pd” and “N_Pd-Pd_error” are coordination numbers and an error in their determination, and “del_E0_Pd-Pd” and “del_E0_Pd-Pd_error” are an energy shift and an error in its determination. The headers “sigma2_Pd-Pd” and “sigma2_Pd-Pd_error” denote the Debye–Waller parameter, its determination error, and “R_Pd-Pd”, “R_Pd-Pd_error”—Pd-Pd interatomic distances and the error of their determination within the coordination sphere Pd-Pd. The following parameters “N_Pd-O”, “N_Pd-O_error”, “del_E0_Pd-O”, “del_E0_Pd-O_error”, “sigma2_Pd-O”, “sigma2_Pd-O_error”, “R_Pd-O”, “R_Pd-O_error” have similar meanings but are expressed by fitting for the Pd-O coordination sphere. The columns with the headings “Oxide_fraction” and “Oxide_fraction_error” indicate the concentration of the oxide phase and should be considered only if oxygen is present in the experiment.

**Figure 6:**
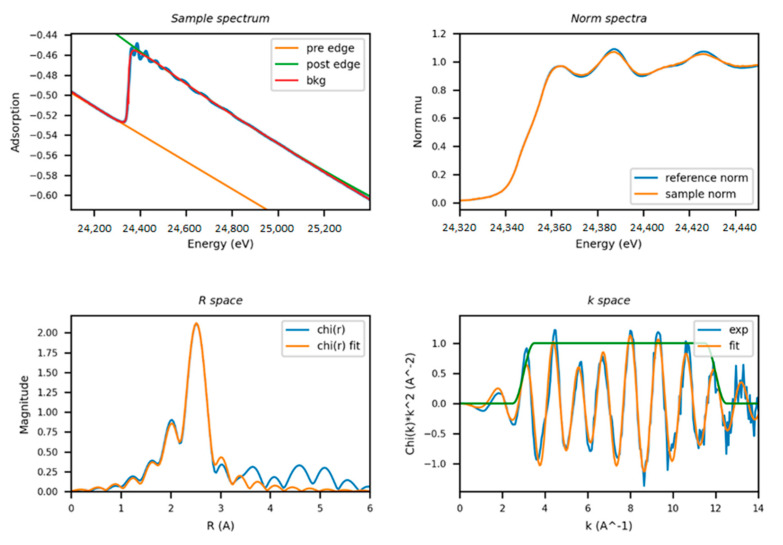
Example of XAS spectra for Pd nanocatalyst in experiment.

The data of the conducted experiments are presented in the form of XAS data, for example, in Figure 6, where k is the wavenumber; A ^ −1 (A^−1^)—dimension (inverse angstroms); Chi is the EXAFS conversion function.

To bring to a convenient format for operating with the data, the values of the spectra were approximated and interpolated using the principal component analysis, the output has obtained the reference values of the normal distribution of the absorption coefficient relative to the energy for each experiment in the series.

### 3.2. Software Implementation

**Figure 7:**
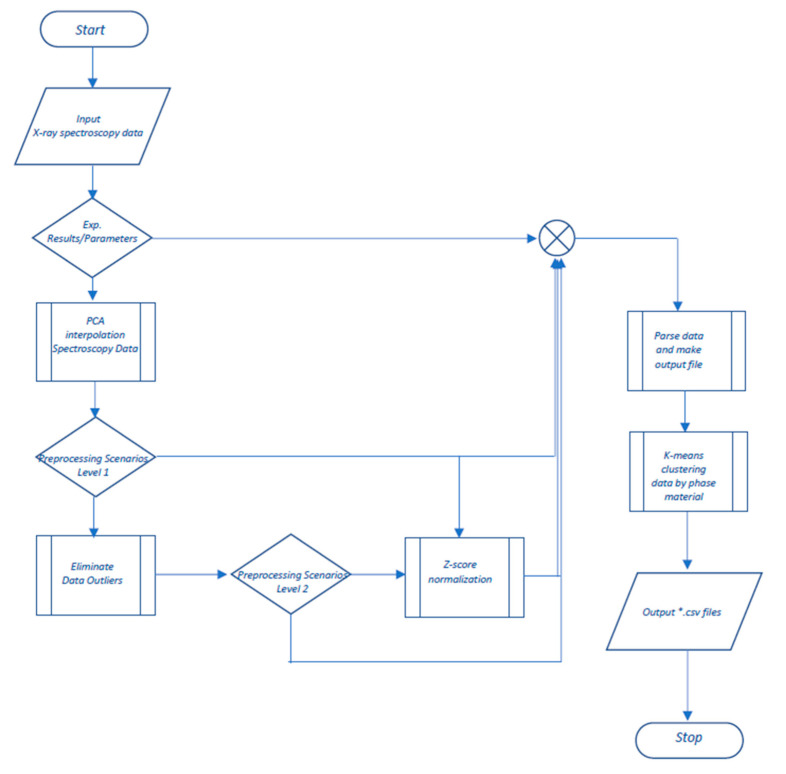
Flow diagram of implemented XAS data preprocessing software.

To simplify the data preprocessing procedure, automated software in C# language was created, which allows you to quickly cope with routine repetitive operations. This language belongs to the object-oriented, high-level structures. When performing computational tasks, it demonstrates an average speed [40]. In addition, it has wide integration capabilities, which can be useful when developing a project into service, in the future, and since the functions of calling and working with equipment provide for low-level APIs, so this choice is a convenient option for working with threads and components, calling the assembler firmware directly. The main functionality of the software includes a preliminary selection of the range of experimental results, formed by the energy level. The algorithm of the proposed software toolkit for preprocessing the XAS data from experiments is quite voluminous; therefore, the flow diagram of the algorithm is given in Figure 7.

Thus, if within each observation there are values that lie outside the range of 24,350–24,950 eV, they are cut off [41], such an optimization of the obtained data allows us to reduce the target materials. In fact, the EXAFS spectrum region remains, which allows one to obtain quantitative information on the size and structure of catalyst nanoparticles, details of the core–shell architecture, and also to track bond lengths both in statics and in dynamics [38]. In addition, there are many methods for analyzing such data, which differ depending on the purpose of the requested information [38]. Furthermore, if necessary (optionally), two stages are performed, and the user makes the choice is made by the user based on the context of the further use of the collected data set. In special cases, with poorly prepared experimental samples, it may be necessary to identify and eliminate outliers in the collected data. To avoid this effect, one of the statistical methods was implemented, in particular the interquartile, since the provided data have a Gaussian distribution [41] the algorithm of the software implementation is given in part (b) in Figure 3.

For the possibility of simple reuse of the obtained data sets, a normalization procedure was implemented using the Z normalization (means) method, which allows you to bring the data values in the input vector to the required range. Basically, this normalization method was added to consider the further use of datasets in solving ML problems in the field of materials science. This method was chosen because it gives relatively good results when processing spectral data [42].

**Figure 8:**
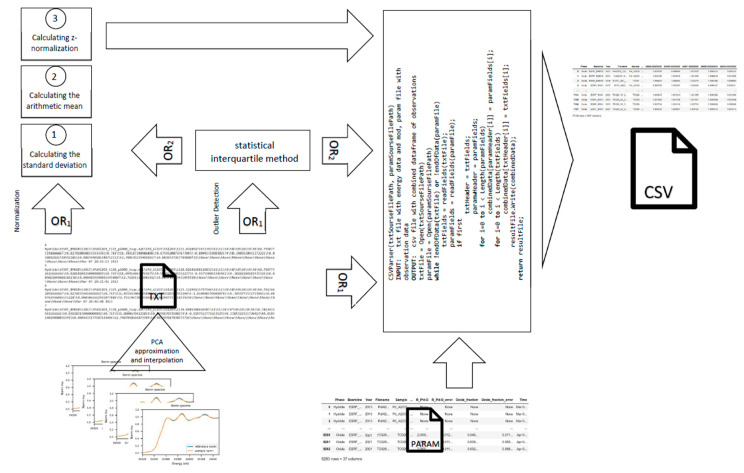
Process of implemented XAS data preprocessing software.

After completing or skipping optional steps, the files containing the parameters and results of the experiment are compared—it is obligatory to find the files within the same directory. If the file comparison is successful, a merge occurs. The parameter headings are followed by the energy levels in the 1 eV step. The results of the obtained data of a specific physical experiment are put in accordance with the set of parameters of this experiment. The output file format is converted to comma-separated values, that is, to * CSV, with a tab added, where the separator is “,”. This format was chosen based on its convenient human interpretation, since in fact, it is textual, widespread, and compatible with almost any data management tool and in general, which characterizes it as a good choice for research analysis [43]. A process of implemented software for XAS data preprocessing is presented in Figure 8.

**Figure 9:**
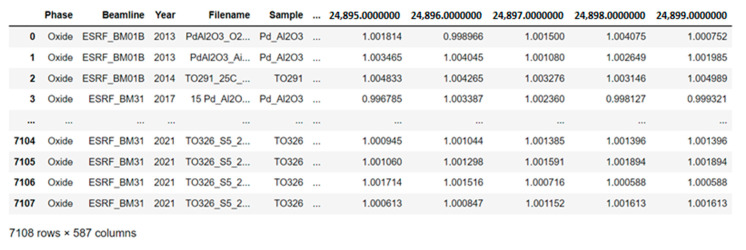
Data frame of processed after XAS experiments with nanocatalysts to study an oxide phase state of Pd nanoparticles.

In addition, four files are created, the key parameter for separation is the phase state of the investigated substance. When new experimental data appear, it is possible to record according to the structural mark of the year. An example of the preprocessed XAS data frame which is ready for further use of ML analysis and prediction is shown in Figure 9.

## 4. Discussion

As a result, based on the obtained data frames of physical experiments based on palladium nanocatalysts using synchrotron radiation, it is possible to operate with different volumes of connected data, for example, within subsets or even within one dimension in a series, depending on the required features of the subject area of research. Values were mapped to determine which X-ray absorption characteristics need to be extracted. The structure of the data obtained within one observation from a series of experiments makes it possible to evaluate the image of the dynamics of changes in the behavior of the material within the limits of a specific continuous change in the parameters of the influence of the external modeling environment. In addition, the K-means machine learning algorithm was implemented, which makes it possible to clarify the quantitative ratio of the phase of the studied material sample at certain types of chemical reactions by marking in cases where the hidden dependencies in the experimental data allow ponder on the presence of a sufficient amount of material in the phase not specified in the initial dataset. Despite the fact that in the received data frames there is an imbalance in the categories of experimental data according to the type of sample under study, gases used for sorption, etc. They provide a good starting point for further accumulation and intelligent post-analysis of data, including the application of ML models as part of highlighting hidden dependencies for their reuse, as well as speeding up the procedure for discovering new functional materials. Thus, the novelty of this paper lies in the creation of software toolkits for XAS data preprocessing with the presentation of results in a form convenient for machine learning, the possibility of reuse, and reproducibility of results. A new set of data has been obtained for research in the field of diagnostics of nanocatalysts.

## 5. Conclusions

Our study was motivated by several research problems: the design and implementation of software tools and preprocessing methods for the enhancement of the experimental XAS data reuse to save research resources; reproducibility of results; also saving time when using the results for subsequent high-quality data mining uses machine learning.

One of the most important results of our research is software for automation of data preprocessing and it is prepared for further data analysis of physical experiments on catalysts based on palladium nanoparticles using synchrotron radiation and creation the de-posited data frame on the results of a series of physical experiments on diagnostics of new functional nanomaterials carried out based on the research accelerator complex, a source of synchrotron radiation in France for the time period 2013–2021. The results presented for consideration will simplify the initial stage of data preparation. These results improved data dissemination and reuse opportunities and improved their integration into the most popular data mining tools, including their use in machine learning models.

Today, there are many complex software solutions and theoretical approaches to the processing and analysis of XAS data. However, their functionality in most cases is biased towards data analysis in order to extract the values and parameters of the materials under study, which does not include detailed approaches to preprocessing and preparing data, which was described in the proposed material. The data preprocessing approach was proposed, in which the output was received with fully ready-made datasets that can save a lot of research resources when they are reused, as well as when they are used to train machine learning models. Representation of the received datasets can transform each individual experiment data point into feature vectors of the equal dimension, which can be useful when using convolutional neural networks. Predictive machine learning models can be used both within the consideration of one phase of the material, and in the full amount of experimental data to predict the absorption coefficient at the corresponding fall energy level. Multiclass classification models will allow missing values to be filled in the experiment protocol.

## Data Availability

The following are available online at https://github.com/codeConcil/Sfedu_csv_parser (accessed on 11 December 2021).
